# Factors Associated with High Sugary Beverage Intake Among Children in Louisiana: A Survey of Caregivers in New Orleans and Baton Rouge

**DOI:** 10.3390/nu17050799

**Published:** 2025-02-26

**Authors:** Melissa Fuster, Yin Wang, Charles Stoecker, Donald Rose, Lisa P. Hofmann, Annie Pasterz, Megan Knapp

**Affiliations:** 1 Department of Social Behavioral and Population Sciences, Tulane University Celia Scott Weatherhead School of Public Health and Tropical Medicine, New Orleans, LA 70112, USA; diego@tulane.edu (D.R.); apasterz@tulane.edu (A.P.); 2 Department of Health Policy and Management, Tulane University Celia Scott Weatherhead School of Public Health and Tropical Medicine, New Orleans, LA 70112, USA; ywang119@tulane.edu (Y.W.); cfstoecker@tulane.edu (C.S.); 3 Department of Public Health Sciences, Xavier University of Louisiana, New Orleans, LA 70125, USA; lhofmann@xula.edu (L.P.H.); mknapp@xula.edu (M.K.)

**Keywords:** child nutrition, sugar-sweetened beverages, restaurant use, survey, parents/caregivers, United States of America (USA)

## Abstract

**Background/Objectives**: Sugar-sweetened beverage (SSB) consumption is associated with child obesity, an understudied issue in the southern United States, where obesity rates are the highest in the country. We examined the factors associated with high SSB intakes among children aged 2–12 years in two major cities in Louisiana, New Orleans and Baton Rouge. **Methods**: We conducted a cross-sectional study using an online survey. The sample consisted of caregivers of children aged 2–12 years who eat restaurant meals (either dine-in, delivery, or take-out) at least once a month and reside in or near New Orleans or Baton Rouge, LA. Multivariable logistic regression was used to examine factors associated with high child SSB intake frequency (≥4 times/week), including restaurant use, caregiver attitudes towards SSB, and their demographics (n = 1006). **Results**: Most caregivers reported weekly child SSB consumption (74.6% ≥ 1×/week; 38.1% ≥ 4+/week) and restaurant use (58.8% ≥ 1×/week). High SSB frequency (≥4+/week) was associated with a higher frequency of restaurant use, lower caregiver education, agreement with the statement that SSBs are an important part of family meals, and disagreement with the statement that restaurants should not offer SSBs with children’s meals (*p* < 0.05). **Conclusions**: Our results revealed a high frequency of SSB consumption among children who dine at restaurants monthly, with significant associations observed between SSB intake, restaurant meals, and pro-SSB attitudes. These findings may support the need for regulations, such as healthy default beverage policies for children’s menus, to potentially reduce SSB intake and shift social norms, particularly in regions with high childhood obesity rates like Louisiana and the southern USA.

## 1. Introduction

Sugar-sweetened beverages (SSBs), which include sodas, fruit drinks, sports drinks, and sweetened waters, teas, and coffees, are the largest source of added sugar in children’s diets and are associated with weight gain and obesity [1,2]. Almost two-thirds of children in the United States (USA) consume at least one SSB per day, with the highest intake among Black, Mexican-American, and non-Mexican Hispanic children and children from low-income families [3,4]. Reduction of SSB intake can decrease the risk of obesity and related diseases [5]. Childhood obesity is disproportionally high within some subpopulations of the USA, with higher and statistically significant prevalences among non-Hispanic Black youth than White youth and among children from families of lower socioeconomic status [6,7]. These issues are of particular concern in southern USA states, as the region has the highest obesity rate in the country (34.1%) [8]. Louisiana has the third-highest childhood/adolescent obesity rate (22.2%, 10–17-year-olds) and the fourth highest among adults (38.1%) [9].

Restaurants, encompassing full-service and fast-food establishments, are a key environment to influence healthy food and beverage choices, as they are an increasingly prominent source of food and beverages globally. In the USA, an estimated one-third of all calories consumed come from food and beverages acquired away from the home, and almost half of food/beverage dollars are spent on eating outside the home [10]. Nearly half of SSB intake occurs away from home [11], making restaurants a prime environment for reducing consumption. Among children ages 4–19 years, 36% consume food from fast-food restaurants in a given day, with the highest rates among non-Hispanic and Black children [12,13]. Consumption of meals in restaurants, especially combination meals or meals from the children’s menu, is associated with higher SSB intake [14,15]. Research also shows children are more likely to purchase an SSB with a combination meal than as a separate item [15,16]. In restaurant settings, Black and Hispanic individuals purchased more beverage calories and grams of sugar per capita than White individuals, with the highest amounts purchased by non-Hispanic Black adolescents and young adults [15].

Past research concerning SSB intake frequency and parental characteristics (e.g., education, gender, age, race/ethnicity, and income) has shown that parents and caregivers are critical in controlling and influencing child food practices at home and at restaurants through modeling food consumption behavior and facilitating food access [17,18,19,20]. Research shows that caregiver attitudes about SSB intake among children, particularly a favorable view towards SSBs, can be a strong factor influencing child SSB intake [17]. Policies, such as healthy default beverage ordinances, and educational interventions may be important to support caregivers in making healthier beverage selections for their children.

There is a paucity of research specifically addressing the southern United States, a region with the highest rates of obesity in the country and where sweetened beverages are important staples in local food cultures [21]. Addressing this need, the objective of this manuscript was to examine SSB intake frequency among children 2–12 years of age, caregiver attitudes, and associated demographic characteristics to inform interventions to reduce SSB consumption.

## 2. Materials and Methods

Our data collection included New Orleans and Baton Rouge, the two largest cities in Louisiana. Participants’ eligibility was determined based on initial screening questions. Inclusion criteria included being an adult (18 years or older), living in or near the target city (New Orleans, LA, USA or Baton Rouge, LA, USA), being a parent or caregiver to a child between the ages of 2–12, and having ordered from or eaten at a restaurant with their children in their city of residence within the last 30 days. We selected the age range, as most children’s menus are marketed to children up to 12 years of age. The surveys were conducted in September 2022 and distributed using an online marketing research firm (Centiment, Denver, CO, USA), which has access to a diverse pool of respondents. The company recruits participants using social media platforms and affiliate networks. Potential participants received invitations to participate that included minimal study information to avoid selection bias. The surveys also utilized fingerprinting technology to ensure unique participants completed the survey and applied measures (e.g., attention checks and response validation) to ensure panelists are fully engaged [22]. Participants who completed the survey were compensated directly by Centiment. A total of 1006 participants consented to participate and completed the survey (506 in New Orleans, LA and 500 in Baton Rouge, LA, USA). These sample sizes allow us to estimate proportions with 5% precision when the confidence level is 95% for each city. This calculation assumes a 20% non-response rate and allows for a Bernoulli distribution with the highest possible variance (i.e., a proportion of 0.5).

Data were collected using online surveys administered via Qualtrics. The survey was developed to be completed within 15 minutes. The survey included a landing page providing information about the study and participation. The survey questions were developed based on past research [23,24,25]. Before full implementation, the survey underwent two pilot tests. First, we shared the survey with a small convenience sample of caregivers (n = 10), to assess how the questions were interpreted by the target audience, ensure clarity, and confirm the estimated completion. Second, before the full distribution, the survey underwent a soft launch with 20 participants as a second verification of the survey questions and to confirm the survey logic was working as intended.

Children’s SSB consumption frequency was collected via the following question: “Thinking about the last 30 days (last month), in general, how often does your child or children consume sugar-sweetened drinks? Please include sodas/pop/carbonated drinks, fruit drinks (Hi-C), sweetened/flavored milk drinks, etc.”, with responses ranging from once per month or less to once a day or more. Following previous research, we categorized intakes of four or more times a week as high [26], generating a dichotomous outcome indicator to explore factors associated with high child SSB intake.

The main explanatory variables in the study were the frequency of restaurant use, attitudes concerning sugary beverages, and selected demographic characteristics. Restaurant use was assessed via the following question: “Thinking about the past 30 days, how often have your children (or child) eaten meals from restaurants? (Please include meals consumed at a restaurant or purchased at a restaurant as take-out or delivery)”. Frequency choices ranged from once a month to more than once a day.

We questioned caregivers about two different attitudes using the following phrases: “Sugary-sweetened drinks are an important aspect of family meals” and “Restaurants should not offer sugar-sweetened drinks with children’s meals”. Responses applied a 5-point Likert scale for agreement, and responses were collapsed into three categories for analysis (disagree, neutral, agree). Lastly, we collected information on basic demographic characteristics, including gender, household income, race/ethnicity, and education level. The gender questions included non-binary categories (trans, non-conforming/gender variant). However, given the small count of some responses, gender was ultimately assessed using a dichotomous variable (female/non-female). Income was asked at the household level, and the responses were collapsed to denote households with incomes above or below USD 50,000, which was the closest data cut to the 2022 median household income in Louisiana of USD 58,330 [27]. We asked participants to identify their racial and ethnic background, with responses collapsed into three categories to denote Black, White, and other/multi-racial for analysis, based on data distribution. Education level was assessed as the highest level completed and collapsed into 3 categories for the multivariable runs: high school graduate or less, some college, and bachelor’s degree or higher, to limit the model specification to categories sufficiently large for statistical inference.

Statistical analyses were conducted using Stata version 16 (StataCorp LLC, College Station, TX, USA), and a threshold of *p* < 0.05 was used to determine statistical significance for all estimates. Multivariable ordered logistic regression models were estimated for each of the three survey questions (frequency of restaurant use, attitude toward SSB importance in family meals, and attitude toward restaurants not offering SSBs with children’s meals) to explore the associations between caregivers’ responses to these survey questions and their sociodemographic characteristics, including gender, race/ethnicity, educational attainment, and income levels, excluding cases with missing data. Respondents that selected “Prefer not to answer” for these variables were set to missing.

Furthermore, a series of multivariable logistic regression models were employed to assess factors potentially associated with SSB intake of children, as denoted by a dummy indicating whether children consumed SSBs at least four times per week. These factors included caregivers’ gender, race/ethnicity, educational attainment, income levels, restaurant use, caregivers’ attitudes toward SSB importance, and their attitudes toward the statement that restaurants should not offer SSBs. As a sensitivity analysis, we also estimated bivariate odds ratios of the associations between attitudes and SSB uptake.

## 3. Results

### 3.1. Sample Characteristics

Table 1 displays the sociodemographic characteristics of the participating caregivers, closely split between New Orleans and Baton Rouge. Most respondents self-identified as female, while more than half self-identified as White. Approximately one-third of the sample had a high school degree or less, with the rest having attended at least some college or more. Additionally, close to half of respondents reported household incomes of less than USD 50,000 per year (Table 1).

Responses varied regarding restaurant use frequency and agreement with statements concerning SSBs. Within our sample, more than half reported consuming meals from restaurants on a weekly basis (Table 1). Nearly half of the respondents disagreed with the statement, “Sugar-sweetened beverages are an important aspect of family meals” while two in five respondents disagreed that “Restaurants should not offer sugar-sweetened drinks with children’s meals” (Table 1).

### 3.2. Factors Associated with Restaurant Use and Attitudes About SSBs

Several socio-demographic characteristics were found to be associated with restaurant use frequency and attitudes concerning SSBs (Table 2). Restaurant use was significantly associated with caregiver gender and income. Respondents who reported higher income and did not self-identify as female were more likely to report higher restaurant use compared with their counterparts (Table 2). Concerning SSB-related attitudes, we found significant associations with gender and race/ethnicity. Concerning the statement that SSBs are important parts of family meals, the level of agreement was significantly associated with gender, race/ethnicity, and education. Compared with their counterparts, female caregivers and those with some college (compared with those with high school or less) were less likely to agree with the statement. On the other hand, those self-identifying as Black were more likely to agree, compared to their White counterparts (Table 2). We also found gender and race/ethnicity to be significantly associated with the attitude that restaurants should not offer SSBs with children’s meals. Higher likelihood of agreement was found among respondents not self-identifying as female and among those self-identifying as Black, compared with those self-identifying as white (Table 2).

### 3.3. Factors Associated with High Sugary Beverage Intake

In general, 38% of the sample reported an overall child SSB consumption frequency of four or more times per week (Table 1). High intake frequency was significantly associated with more frequent restaurant use. Compared with those using restaurants monthly, caregivers who dined in restaurants weekly were about 49% more likely to report high SSB intake of their children, while those who dined in restaurants twice or more per week had twice the likelihood of reporting high child SSB intake (Table 3). The associations between caregivers’ attitudes toward the two SSB-related statements and their children’s SSB intake frequency were essentially aligned. High SSB consumption was more likely to be found among children whose caregivers were neutral or agreed that SSBs were an important part of family meals, while high consumption was less likely to be reported by caregivers who stayed neutral or held views that restaurants should not offer SSBs with children’s meals (Table 3). Regarding the sociodemographic characteristics, caregivers with the highest education level (bachelor’s degree or higher) were less likely to report high SSB intake among their children compared with those with the lowest education level (high school or less) (Table 3).

## 4. Discussion

High child SSB intake frequency (4+ times a week) was reported by 38.1% of caregivers of children between the ages of 2–12 included in the study. This is higher than what has been reported in a previous analysis of the 2021 National Survey of Children’s Health where high intake was found among 21% of respondents. However, the National Survey included younger children (1–5 years old) in their sample, and older children in that sample (4 and 5-year-olds) showed higher intake frequencies (27.7% and 25.6%, respectively) [26]. High SSB intake was significantly associated with parental education. This association has been documented in previous research [26,28,29]. While past research has documented greater overall frequency of SSB consumption among Black and Hispanic families, compared to Non-Hispanic White, this study found no significant association between child SSB intake frequency and race/ethnicity [26,29]. This may be the result of our sample distribution, where more than half of the caregivers self-identified as White, but an additional explanation may be the context of our work. Louisiana is among the states with the highest SSB intake in the country, where 68% of adults consume at least one SSB per day [30]. The southern US region presents the highest proportions of adults that consume SSBs on a daily basis [31], a trend that concurs with the region’s historical preference for sweet and soft drinks, with many of today’s leading beverage brands tracing their roots back to the region [21].

Our findings concerning the positive association between child SSB intake frequency and restaurant use concur with past research [11,14]. We found that restaurant use with children was significantly associated with income and gender, that is, lower use was found among respondents with lower income and self-identifying as female. Research examining gender and feeding behaviors has shown that fathers tended to eat out more when feeding their children, and such eating occasions were significantly and positively associated with SSB consumption [20]. While gender was not significantly associated with overall child SSB intake frequency in this study, future research may examine differences in beverage choice and amount within restaurant meals to explore this association further.

Our study also underscored the importance of social norms concerning SSBs in family meals and restaurant meals, which supports what past research has shown. Woo Baidal et al. reported that negative attitudes toward SSBs were associated with lower consumption, and these associations remained after adjusting for age and race/ethnicity [19]. While our results showed no significant associations in child SSB intake frequency by caregiver race/ethnicity, caregivers identifying as Black were more likely to perceive SSBs as important parts of family meals. This perception was also associated independently with lower educational attainment.

A higher percentage of our sample disagreed with the statement that restaurants should not offer SSBs with children’s meals—a factor potentially hinting at resistance towards restaurant/business regulations, such as the requirement for restaurants to offer healthy beverages as the default choice with these meals. While some past work has documented positive attitudes towards policies that promote healthier choices in restaurants, these were documented among restaurant-initiated changes, such as the voluntarily adopted LiveWell initiative [32]. USA-based research examining public attitudes about healthy eating policies documents how this issue is seen as one of personal responsibility, and this view is linked to opposition to government regulation [33].

While our study contributes to the growing research on the influence of restaurant meals on children’s dietary quality, in an under-researched population, our results interpretation must account for some limitations. First, our sample is not representative of the broader population in the state or region. It specifically focused on caregivers who report eating at restaurants at least once a month and was limited to participants from the two largest cities in the state. Results may be different from caregivers with less frequent restaurant use or living in less urban settings. Consideration should also be given to inherent limitations of self-report and recall bias, which may lead to over- or under-reporting SSB consumption and restaurant use. While our results largely concur with past research, future work can address the association between restaurant use and SSB intake among children in more rural settings, with a lower density of restaurants. Our study did not capture interpersonal variables that may influence SSB frequencies, including children’s pestering behavior and exposure to marketing [17]. Past research has also shown that SSB frequency varies by child age and gender, and parental age [17,26,29], which are variables not addressed in the current analysis.

## 5. Conclusions

This research focused on an understudied population in a region with one of the highest SSB intakes in the USA. The results have the potential to support the implementation of restaurant-based policies to address SSB consumption, including a recently implemented healthy default beverage ordinance in New Orleans, LA, USA. This policy requires restaurants to offer healthy default beverages (water, low or non-fat milk, 100% juice) with children’s meals [34], with the possibility to tackle children’s SSB consumption in restaurants. Policies like this may have the potential to facilitate healthier choices and change social norms around child beverage consumption. At the same time, our work also underscores the need to independently address social norms, through public education campaigns, since they clearly influence SSB consumption, and may do so even in the presence of regulations that require restaurants to offer healthier default options for children’s meals.

## Figures and Tables

**Table 1:** Sample characteristics (n = 1006).

Characteristics		Overall	
		n	%
Location	New Orleans	506	50.3%
	Baton Rouge	500	49.7%
Gender	Female	741	73.7%
	Male	259	25.7%
	Trans female	1	0.1%
	Non-conforming/gender variant	5	0.5%
Race/Ethnicity	Asian	14	1.4%
	Black/African American	302	30.0%
	Latin/Hispanic	17	1.7%
	Native American	11	1.1%
	NH/Pacific Islander	2	0.2%
	White	596	59.2%
	Multiracial	64	6.4%
Education Level	Less than high school	24	2.4%
	High school/GED	268	26.6%
	Some college	329	32.7%
	Bachelor’s degree	248	24.7%
	Post-graduate education	137	13.6%
Annual Household Income	<USD 9999	78	7.8%
	USD 10,000–USD 24,999	141	14.0%
	USD 25,000–USD 49,999	248	24.7%
	USD 50,000–USD 74,999	181	18.0%
	USD 75,000–USD 99,999	130	12.9%
	USD 100,000–USD 149,999	136	13.5%
	USD 150,000 and greater	61	6.1%
	Prefer not to say	31	3.1%
Child SSB Intake Frequency	1+/day	255	25.3%
	4–6/week	128	12.7%
	2–3/week	252	25.0%
	1/week	116	11.5%
	2–3/month	146	14.5%
	1/month or less	109	10.8%
Restaurant Use Frequency	1/month	142	14.1%
	2/month	272	27.0%
	1/week	277	27.5%
	2–3/week	256	25.4%
	4+/week	59	5.9%
SSBs are an important part of family meals	Disagree	481	47.8%
	Neutral	326	32.4%
	Agree	199	19.8%
Restaurants should not offer SSBs with children’s meals	Disagree	413	41.1%
	Neutral	369	36.7%
	Agree	224	22.3%

Abbreviations: SSBs: Sugar-sweetened beverages.

**Table 2:** Caregiver sociodemographic characteristics associated with restaurant use and caregiver attitudes about sugar-sweetened beverages.

Covariates:	Restaurant Use		SSB are Important in Family Meals		Restaurants Should Not Offer SSB with Children’s Meals	
	OR	[95% CI]	OR	[95% CI]	OR	[95% CI]
Gender						
Non-Female	REF		REF		REF	
Female	0.741 *	[0.568, 0.967]	0.497 **	[0.378, 0.653]	0.647 **	[0.493, 0.850]
Race						
White	REF		REF		REF	
Black	1.133	[0.861, 1.491]	1.681 **	[1.276, 2.214]	1.636 **	[1.244, 2.151]
Other/Multiracial	1.010	[0.692, 1.475]	0.935	[0.631, 1.385]	1.205	[0.825, 1.761]
Caregiver educational attainment						
High school or less	REF		REF		REF	
Some college	1.289	[0.948, 1.751]	0.689 *	[0.507, 0.935]	0.837	[0.621, 1.129]
Bachelor’s degree or higher	1.300	[0.942, 1.795]	0.989	[0.715, 1.368]	1.133	[0.821, 1.565]
Annual household income						
≤USD 49,999	REF		REF		REF	
≥USD 50,000	1.691 **	[1.284, 2.227]	0.954	[0.721, 1.264]	0.93	[0.705, 1.227]
n	975		975		975	

Abbreviations: OR: Odds ratios for adjusted (multivariate) model for ordered logistic regressions; REF, reference, which denotes the reference category of the regression estimates for categorical variables; SSB, sugar-sweetened beverage. Notes: Each column is a separate multivariate ordered logistic regression model for three different outcomes. The order of the outcome “restaurant use” is restaurant use monthly (level 1), restaurant use weekly (level 2), and restaurant use 2×/week (level 3). The order of the outcome “attitude about SSB importance” disagrees with the statement “SSBs are an important aspect of family meals” (level 1), neutral about the statement (level 2), and agree with the statement (level 3). The order of the outcome “attitude about the statement that restaurants should not offer SSBs” disagrees with the statement (level 1), neutral about the statement (level 2), and agree with the statement (level 3). Odds ratios are followed by 95% confidence intervals in square brackets. Significance levels: * *p* < 0.05, ** *p* < 0.01.

**Table 3:** Factors associated with high children’s sugar-sweetened beverage intake frequency (4+/week).

	Child High SSB Intake (4+/week)	
Covariates	Adjusted ORs	[95% CI]
Caregiver gender		
Non-Female	REF	
Female	1.325	[0.956, 1.838]
Caregiver race		
White	REF	
Black	1.045	[0.757, 1.442]
Other/Multiracial	0.999	[0.628, 1.589]
Caregiver educational attainment		
High school or less	REF	
Some college	0.926	[0.655, 1.308]
Bachelor’s degree or higher	0.584 **	[0.400, 0.854]
Annual household income		
≤USD 49,999	REF	
≥USD 50,000	0.824	[0.598, 1.134]
Restaurant use frequency		
Monthly (≤2 month)	REF	
1/week	1.494 *	[1.058, 2.109]
≥2/week	2.001 **	[1.433, 2.795]
Caregiver level of agreement: “SSBs are an important part of family meals”		
Disagree	REF	
Neutral	1.956 **	[1.428, 2.679]
Agree	1.670 **	[1.139, 2.448]
Caregiver level of agreement: “Restaurants should not offer SSBs with children’s meals”		
Disagree	REF	
Neutral	0.426 **	[0.312, 0.582]
Agree	0.312 **	[0.212, 0.460]
n	975	

Abbreviations: OR: odds ratios for adjusted (multivariate) model; CI: confidence intervals for OR; REF, reference, which denotes the reference category of the regression estimates for categorical variables; SSB, sugar-sweetened beverage. Note: Significance levels: * *p* < 0.05, ** *p* < 0.01

## Data Availability

The original contributions presented in this study are included in the article. Further inquiries can be directed to the corresponding author.
